# KIAA0753 enhances osteoblast differentiation suppressed by diabetes

**DOI:** 10.1111/jcmm.70035

**Published:** 2024-09-08

**Authors:** Mengxue Li, Yongqin Wang, Xiangmei Wu, Quanmei Chen, Jianguo Huang, Huifang Zhu, Shengyong Yang, Jichun Wang, Le Tai Li, Xianjun Liu, Kang Fu, Fangzhou Song, Changdong Wang

**Affiliations:** ^1^ Department of Biochemistry and Molecular Biology, Molecular Medicine and Cancer Research Center, School of Basic Medical Sciences Chongqing Medical University Chongqing China; ^2^ Department of Gastrointestinal Surgery Traditional Chinese Medicine Hospital of Shizhu Chongqing China; ^3^ Department of Physiology, Molecular Medicine and Cancer Research Center, School of Basic Medical Sciences Chongqing Medical University Chongqing China; ^4^ A Division of Providence Cancer Institute Earle A. Chiles Research Institute Portland Oregon USA; ^5^ Sangon Biotech (Shanghai) Co., Ltd. Shanghai China

**Keywords:** diabetes‐related bone loss, Gli2, Hedgehog signalling pathway, KIAA0753, osteoblast differentiation, ubiquitination

## Abstract

Diabetes‐related bone loss represents a significant complication that persistently jeopardizes the bone health of individuals with diabetes. Primary cilia proteins have been reported to play a vital role in regulating osteoblast differentiation in diabetes‐related bone loss. However, the specific contribution of KIAA0753, a primary cilia protein, in bone loss induced by diabetes remains unclear. In this investigation, we elucidated the pivotal role of KIAA0753 as a promoter of osteoblast differentiation in diabetes. RNA sequencing demonstrated a marked downregulation of KIAA0753 expression in pro‐bone MC3T3 cells exposed to a high glucose environment. Diabetes mouse models further validated the downregulation of KIAA0753 protein in the femur. Diabetes was observed to inhibit osteoblast differentiation in vitro, evidenced by downregulating the protein expression of OCN, OPN and ALP, decreasing primary cilia biosynthesis, and suppressing the Hedgehog signalling pathway. Knocking down KIAA0753 using shRNA methods was found to shorten primary cilia. Conversely, overexpression KIAA0753 rescued these changes. Additional insights indicated that KIAA0753 effectively restored osteoblast differentiation by directly interacting with SHH, OCN and Gli2, thereby activating the Hedgehog signalling pathway and mitigating the ubiquitination of Gli2 in diabetes. In summary, we report a negative regulatory relationship between KIAA0753 and diabetes‐related bone loss. The clarification of KIAA0753's role offers valuable insights into the intricate mechanisms underlying diabetic bone complications.

## 1 INTRODUCTION

Diabetes is a chronic metabolic disorder characterized by inadequate insulin secretion or low insulin sensitivity.^1^ Diabetes, companied with high blood glucose, often leads to many complications by inducing oxidative stress at the cellular level,^2^ increasing advanced glycation end products (AGEs)^3^ and reactive oxygen species (ROS),^4^ enhancing the expression of cytokines^5^ and so on. Impaired bone qualities and increased fracture risks have been recognized as complications of diabetes.^6^ These factors affect the function of osteoblasts and osteoclasts, leading to a reduction in bone formation.^7^ Bone tissues are important for mammalian species that allow muscular attachment and support body movement. Osteocytes are the most abundant cells in bone, and their response to mechanical stimulation alters the release of molecules that regulate the function of osteoclasts and osteoblasts, reducing osteocyte apoptosis and promoting bone formation.^8^ Osteoblasts are characterized by generating the richest organic matrix component in bone that alkaline phosphatase (ALP), osteocalcin (OCN) and collagen type I, playing an important role in bone remodelling.^9^ Bone remodelling is a continuously dynamic process throughout a lifetime in which old or damaged mineralized bones are removed by bone resorption cells of osteoclasts, and replaced by a new bone matrix (osteoid) produced by osteoblasts. Then osteoid becomes fully mineralized bone tissue.^10^

Primary cilia are highly conserved organelles that protrude from the surface of almost every cell type in vertebrates, dividing into the ciliary tip, the membrane‐bound axoneme extending from the surface, the transition zone and the basal body complexes.^11^ Primary cilia can sense chemical changes and mechanical stimuli to transduce signalling through the G protein‐coupled receptors,^12^ playing an important role in the transduction of the Hedgehog (Hh) signalling pathway.^13^ The Hh signalling pathway regulates lots of cell fate and the processes of self‐renewal in development and tissue homeostasis.^14^ Defects in the length and morphology of primary cilia often lead to dysregulation of signalling transduction and cell function, which in turn lead to developmental diseases called ciliopathies.^15^ Recent studies suggest that diabetes inhibits the gene expression of primary cilia and primary cilia formation, thereby affecting diabetic fracture healing in osteoblasts.^16^

KIAA0753, a primary cilia protein, regulates ciliogenesis and cilia maintenance.^17^ In humans, mutations in KIAA0753 can cause OFD and Jeune syndrome, with skeletal dysplasia such as narrow thorax, short ribs and cerebellar hypoplasia.^18^, ^19^ Previous reports have shown that the deletion of KIAA0753 leads to bone loss to cause skeletal ciliopathies.^20^ Diabetes can also cause bone loss, however, whether KIAA0753 has a direct effect on bone loss in diabetes is completely unknown.

In this study, we generated diabetes cell models and diabetes rats to confirm the expression of KIAA0753 was downregulated on bone loss in the diabetic microenvironment. The osteoblast differentiation and primary cilia growth were both suppressed by high glucose, but overexpression of KIAA0753 could rescue the inhibition of osteoblast differentiation and primary cilia growth by high glucose. Meanwhile, there was a significant reduction in the length of primary cilia after knockdown of KIAA0753. This process depended on KIAA0753 binding with Gli2, SHH and OCN to activate the Hh signalling pathway directly and reduced the ubiquitination of Gli2 protein in high glucose. These results provide a potential therapeutic target for treating bone loss induced by diabetes.

## 2 MATERIALS AND METHODS

### 2.1 Reagents and antibodies

The following reagents and antibodies were used: α‐ MEM medium (Hyclone), Fetal Bovine Serum (ExCell Bio), Alizarin Red (Solarbio), cetypyridinium chloride (Sigma), Alkaline Phosphatase Kit (Biosion bio‐technology), Cyclopamine (CST), β‐glycerophosphate (Sigma), Penicillin–Streptomycin Solution (Hyclone), dexamethasone (Sigma), ascorbic acid (Sigma), acetylated α‐tubulin antibody (1:1000, T6793,Sigma), γ‐tubulin antibody (1:1000, 10068‐1‐AP, Proteintech), ARL13B (1:1000,17711‐1‐AP, Proteintech), ubiquitination antibody (1:1000, 10201‐ 2‐AP, Proteintech), OPN (1:1000, 225952‐1‐AP, Proteintech), ALP (1:1000, A5111, Selleckchem), SHH (1:1000, A5115, Selleckchem), OCN (1:1000,16157‐1‐AP, Proteintech), SuFu (1:1000, C5462, Selleckchem), KIAA0753 (1:1000, YE3924291B, Invitrogen), Gli2 (1:1000, D262758, Sangon Biotech,), GAPDH (1:1000, D110016, Sangon Biotech), Vinculin (1:2000, 26520‐1‐AP, Proteintech), Flag (1:1000, D110005, Sangon Biotech), Goat‐anti‐raabit IgG (1:1000, D111018, Sangon Biotech), HRP‐linked goat anti‐rabbit IgG antibody (1:5000, D110058,Sangon Biotech), HRP‐linked goat anti‐mouse IgG antibody (1:5000, D110087, Sangon Biotech), Alexa Fluor 568 conjugated anti‐rabbit antibody (Invitrogen), Alexa Fluor 647 conjugated anti‐mouse antibody (Invitrogen), DAPI (Sigma), Lipofectamine® 2000 DNA Transfection Reagent (Invitrogen).

### 2.2 Diabetic cell models construction

Referring to the previous report.^21^ Using MC3T3‐E1 cells to construct the diabetic cell models in vitro that adding D−(+)− glucose in α‐ΜΕΜ media (containing 10% FBS and 1% Penicillin–Streptomycin solution) to simulate a high glucose environment. MC3T3‐E1 cells were induced by α‐MEM media supplemented with D−(+)− glucose to a final glucose concentration of 5.6 mM (NC groups) or 25 mM (HG groups), simulating normal and high glucose conditions, respectively.

### 2.3 RNA sequencing

Whole‐transcriptome sequencing was performed using MC3T3‐E1 treated with high glucose (25 mM) and normal glucose (5.6 mM) for 72 h. Total RNA was extracted by the standard TRIzol extraction method, and cDNA was sequenced using the MGI system by Wuhan Frasergen Bioinformatics Co. Ltd (Shanghai, China). KEGG enrichment analysis, GSEA, and heatmap analysis were performed for all differentially expressed genes. A significant threshold of *p* < 0.05 was applied in KEGG enrichment analysis.

### 2.4 T1DM SD rats model construction

Referring to the methods of Tao et al.^22^ The 8 weeks of SD rats were purchased from the experimental Animal Center of Chongqing Medical University. Then STZ was dissolved in a 50 mM sodium citrate buffer (pH = 4.5) at a final concentration of 100 mg/mL. The SD rats were injected intraperitoneally with a single large dose of STZ (1 mL/kg) to induce diabetes or sodium citrate buffer (1 mL/kg) as a control group. After a week, SD rats with random blood glucose concentrations greater than 250 mg/dL (13.89 mmol/L) were defined as T1DM. All animal experimental procedures were conducted with the approval of the Animal Ethics Committee of Chongqing Medical University and following the related regulations on animal testing and research ethics.

### 2.5 Transfection

MC3T3‐E1 cells or HEK293T cells were seeded on 6‐well plates at 4 × 10^4^ cells/well. When fusion reached 80%, cells were transfected with pcDNA3.1‐Flag plasmids, pcDNA3.1‐KIAA0753‐Flag plasmids, pCS2‐Gli2‐Myc, pcDNA3.1‐OCN‐His or GV358‐SHH‐Flag by using the Lipofectamine 2000 Transfection Reagent to culture for 4 h in serum‐free media and then induced by α‐MEM media for 48 or 72 h.

### 2.6 Western blot

MC3T3‐E1 were homogenized with RIPA (radioimmunoprecipitation assay) buffer containing Protease inhibitors on ice. The proteins were denatured in the 6 × loading buffer and equal amounts of proteins were separated in SDS‐PAGE gels. Proteins were transferred to PVDF membranes in the solution containing 20% methanol. Then blocking in 5% defatted milk for 2 h, incubated with antibody overnight at 4°C and horseradish peroxidase (HRP)‐conjugated antibody at room temperature for 1 h. Then developed with ECL Reagents and visualized was performed using BIO RAD ChemiDoc™ Touch Imaging System (USA). Band intensities were quantified using ImageJ software.

### 2.7 Alizarin red staining

MC3T3‐E1 cells were induced by osteoblast differentiation media (OS media, containing 10% FBS, 1% Penicillin–Streptomycin solution, 10 mM β‐glycerophosphate, 1 × 10^−8^ M dexamethasone and 50 μg/mL ascorbic acid) with different glucose concentrations for 21 days, then stained with 0.2% alizarin red solution (pH 8.3) at room temperature for 1 h and scanned stained cells. Then cells were destained with 10% (w/v) cetylpyridinium chloride for 30 min at 37°C. The 100 μL solutions were transferred to the 96‐well plate and optical density was measured at 562 nm.

### 2.8 ALP activity analysis

ALP activity was detected by the ELISA kit according to the manufacturer's instructions.^23^ After mixing 200 μL working solution, 2 μL cell proteins and 18 μL ddH2O, the solutions were incubated at 37°C for 1 min and measured at 405 nm. Subsequently, we calculated the rate of change of the A value per minute (∆A/min). ALP activity was determined according to the formula (2757 × ∆A/min) U/L.

### 2.9 Immunofluorescence

MC3T3‐E1 cells were induced by 5.6, 25, 25 mM + overexpression KIAA0753, sh‐NC or sh‐KIAA0753 for 3 days. The media were discarded and the cells were washed three times with PBS, then fixed with 4% paraformaldehyde for 10 min. Fixed cells were permeabilized with 0.05% Triton X‐100 for 10 min. Cells were then incubated with 5% BSA to block non‐specific antigen sites for 1 h, and incubated with primary antibodies overnight at 4°C. Alexa Fluor568‐conjugated anti‐rabbit antibodies and Alexa Fluor647‐conjugated anti‐mouse antibodies were used as secondary antibody for 1 h. Observation and photography were performed using a Leica DM4000 microscope.

### 2.10 Construction and transfection of interfering lentivirus

KIAA0753 short hairpin RNA (shRNA) was constructed according to the KIAA0753 sequence (NCBI Reference Sequence: NC_000017.11) published on GenBank. The sense sequence was 5′‐CCGGGTACAGACTTCAAGGATGACTCGAGTCATCCTTGAAGTCTGTACTTTTTTG‐3′, the antisense 5′‐AATTCAAAAAAGTACAGACTTCAAGGATGACTCGAGTCATCCTTGAAGTCTGTAC‐3′. The KIAA0753‐shRNA fragments were inserted into the pLKO.1‐puro lentiviral vector and then transfected into MC3T3‐E1 cells for 72 h for western blot or immunofluorescence.

### 2.11 Co‐immunoprecipitation

HEK293 T cells or MC3T3‐E1 cells were transfected with the plasmids of pcDNA3.1‐Flag, pcDNA3.1‐KIAA0753‐Flag, GV358‐SHH‐Flag, pCS2‐Gli2‐Myc or pcDNA3.1‐OCN‐His by using Lipofectamine 2000 for 48 h, and cells were lysed by RIPA buffer containing PMSF and the lysate separately incubated with the antibody of Flag, KIAA0753, Gli2 or IgG at 4°C for 24 h with gentle shaking. Then antibody–antigen conjugates were captured with Protein A+G Agarose for 24 h with gentle shaking. To discard non‐specific binding proteins, the beads were washed with 1000 μL PBS for 5 min 10 times. The proteins were then boiled and analysed by western blot.

### 2.12 Statistical analysis

All data were presented as mean ± SD (*n* ≥ 3). Student's *t*‐test was employed to compare two groups and one‐way ANOVA was used to compare more than two groups. A significance threshold of *p* < 0.05 was applied. GraphPad Prism was utilized for data visualization.

## 3 RESULTS

### 3.1 Inhibition of osteoblast differentiation and impaired primary cilia biosynthesis under high glucose conditions

**FIGURE 1:**
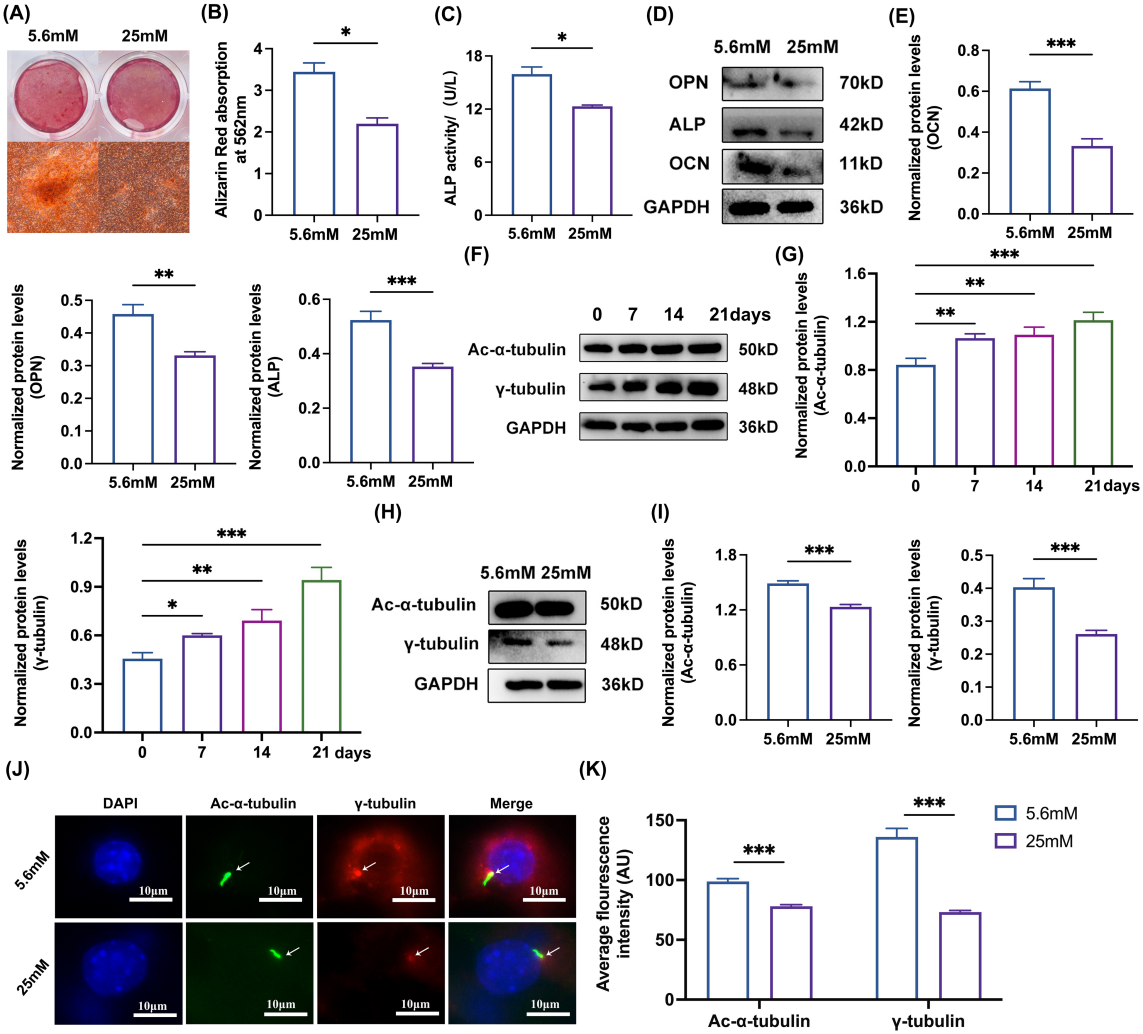
Inhibition of Osteoblast differentiation and primary cilia biosynthesis by high glucose. (A and B) Alizarin red staining and the quantitative analysis of Alizarin red staining at 562 nm in MC3T3‐E1 treated with 5.6 or 25 mM OS media for 21 days. (C) ELISA kit assayed ALP activity in MC3T3‐E1 cells treated with 5.6 or 25 mM OS media for 5 days. (D and E) Western blot of OCN, OPN and ALP expression in MC3T3‐E1 treated with 5.6 or 25 mM OS media for 3 days. (F and G) Western blot of Ac‐α‐tubulin and γ‐tubulin expression in MC3T3‐E1 cells after induced by OS media for 0, 7, 14 and 21 days. (H and I) Western blot of Ac‐α‐tubulin and γ‐tubulin expression in MC3T3‐E1 treated with 25 mM OS media for 3 days. (J and K) Immunofluorescence staining of primary cilia in MC3T3‐ E1 treated with 5.6 or 25 mM OS media for 3 days. Quantitative analysis of average fluorescence intensity of Ac‐α‐tubulin and γ‐tubulin. Blue, DAPI; Green, Ac‐α‐tubulin; Red, γ‐tubulin, scale bar: 10 μm. All the data were represented as means ± SEM. *p < 0.05, **p < 0.01, ***p < 0.001 (*n* = 3).

To explore the effects of diabetes on osteoblast differentiation, we generated diabetic cell models in vitro. Alizarin red staining showed that the formation of mineralized nodules was reduced in HG groups compared with NC groups (Figure 1A,B). Meanwhile, ALP activity of the total cellular protein assayed by the ELISA kit showed a decrease in HG groups (Figure 1C). We also noted that the expression of osteoblast differentiation marker protein OCN, OPN and ALP all decreased in HG groups compared with NC groups (Figure 1D,E). To explore the relationship between osteoblast differentiation and primary cilia biosynthesis, MC3T3‐E1 cells were induced with osteogenic media for 0, 7, 14 and 21 days to assess the expression of primary cilia markers, Ac‐α‐ tubulin and γ‐tubulin. Results showed an increase in Ac‐α‐tubulin and γ‐tubulin expression during osteoblast differentiation (Figure 1F,G). To investigate whether diabetes affects primary cilia biosynthesis, we observed decreased expression of Ac‐α‐tubulin and γ‐tubulin in HG groups compared with NC groups (Figure 1H,I). Furthermore, a morphological analysis of primary cilia in MC3T3‐E1 cells revealed shorten cilia length in HG groups compared with NC groups (Figure 1J,K). These results indicated that primary cilia biosynthesis was decreased in diabetes, leading to the inhibition of osteoblast differentiation and mineralization.

### 3.2 Decreased expression of KIAA0753 under high glucose conditions: insights from in vitro and in vivo analysis

**FIGURE 2:**
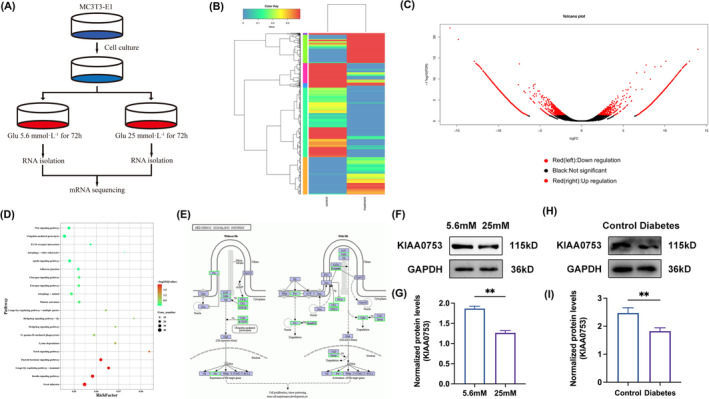
Significant decrease in KIAA0753 expression under high glucose in vitro and in vivo. (A) Experimental design and procedure for sample preparation for RNA sequencing. (B) Heatmap showing differentially expressed genes between HG groups and NC groups. Red: High expression levels. Blue: Low expression levels. (C) Volcano plot illustrating upregulated (right red dots) and downregulated (left red dots) genes induced by high glucose. (D) KEGG pathway enrichment analysis of the differentially expressed genes. Red: High expression levels. Green: Low expression levels. (E) KEGG database visualization of Hedgehog signalling pathway associated genes affected by high glucose. (F and G) Western blot of KIAA0753 expression in MC3T3‐E1 cells treated with 5.6 or 25 mM OS media for 3 days. (H and I) Western blot of KIAA0753 in control rats and diabetes rats. All the data were represented as means ± SEM. **p < 0.01 (*n* = 3).

To further investigate the mechanisms of diabetes‐related bone loss, we performed RNA sequencing to determine differently expressed genes between the NC groups and HG groups. The experimental design and procedure for establishing the diabetes cell models were illustrated in Figure 2A. Employing criteria of a differential multiple >2 and a false discovery rate <0.05 (or 0.01), we identified a total of 4363 differentially expressed genes, with 1808 genes upregulated and 2555 genes downregulated (Figure 2B,C). Notably, the gene KIAA0753 exhibited significant downregulated in HG groups compared to NC groups. Subsequently, Kyoto Encyclopedia of Genes and Genomes (KEGG) pathway enrichment analysis based on RNA sequencing data, highlighted a substantial alteration in the Hh signalling pathway by primary cilia had a significant change (Figure 2D). The identified genes associated with the Hh signalling pathway were visually represented in Figure 2E. Then we performed western blot analysis to confirm a significant decrease in KIAA0753 protein expression in the diabetic cell models (Figure 2F,G), aligning with the RNA sequencing results. To further corroborate our findings in an in vivo context, we established diabetic mouse models and extracted bone protein from the mouse femur. The data demonstrated a substantial decrease in KIAA0753 expression in diabetic mouse (Figure 2H,I). These collective results strongly suggested a negative relationship between KIAA0753 expression and diabetes‐related bone loss.

### 3.3 Temporal increase in KIAA0753 expression during osteoblast differentiation and KIAA0753's cellular localization

**FIGURE 3:**
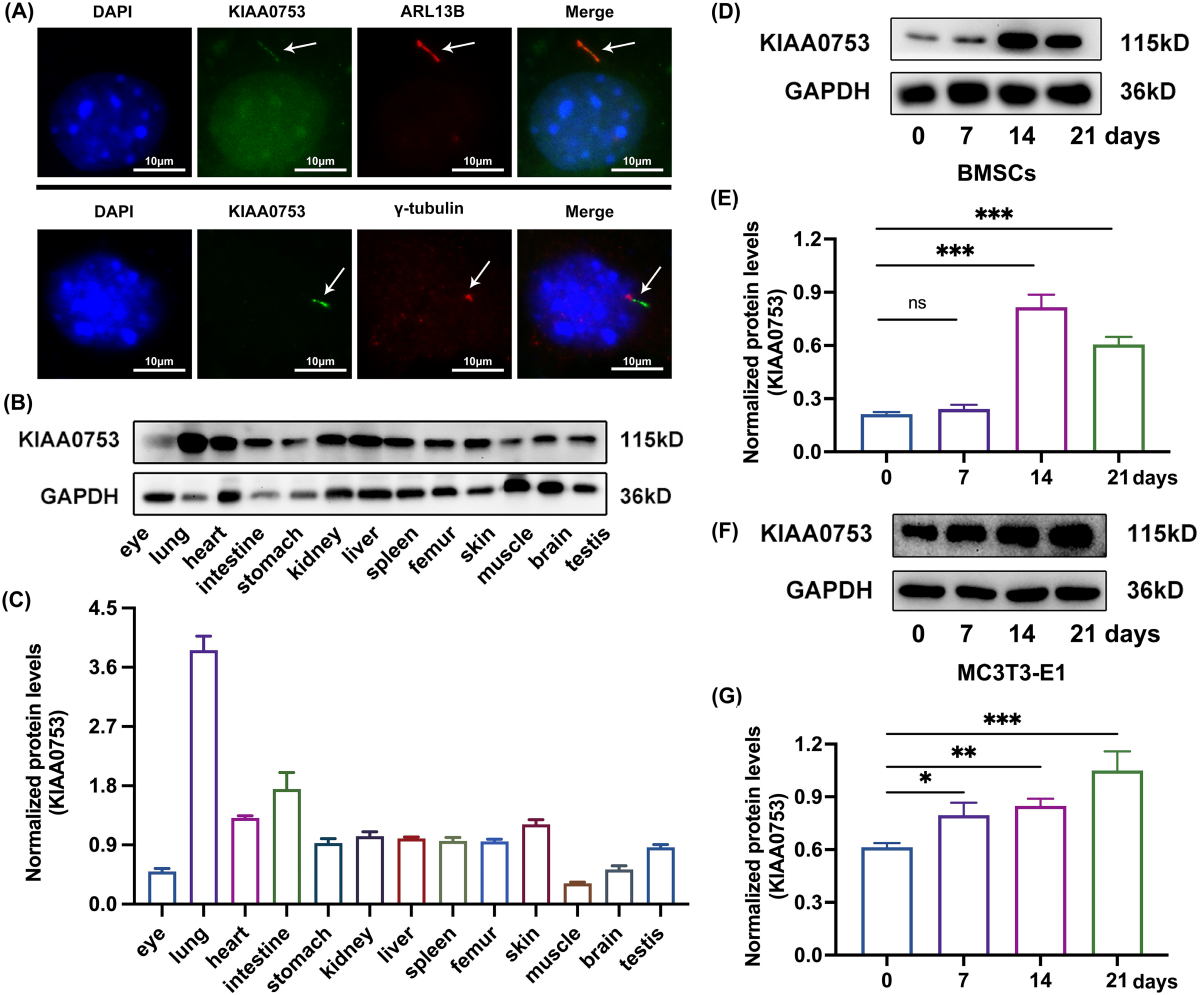
KIAA0753's location and its time‐dependent expression pattern during osteoblast differentiation. (A) Immunofluorescence staining of primary cilia and KIAA0753 in MC3T3‐E1. Blue, DAPI; Green, KIAA0753; Red, ARL13B and γ‐tubulin; scale bar = 10 μm. (B and C) Western blot of KIAA0753 expression in the eye, lung, heart, intestine, stomach, kidney, liver, spleen, femur, skin, muscle, brain and testis of the mouse. (D and E) Western blot of KIAA0753 expression in BMSCs after induced by OS media for 0, 7, 14 and 21 days. (F and G) Western blot of KIAA0753 expression in MC3T3‐E1 after induced by OS media for 0, 7, 14 and 21 days. All the data were represented as means ± SEM. *p < 0.05, **p < 0.01, ***p < 0.001, ns, no significance (*n*=3).

To delineate the cellular localization of KIAA0753, we analysed its distribution in MC3T3‐E1. Immunofluorescence staining revealed a specific distribution of KIAA0753 exclusively along the axis of primary cilia, with no presence observed in the basal body (Figure 3A). Further investigation of the tissue‐specific expression patterns of KIAA0753 encompassed an examination across 13 different mouse tissues. Results indicated high expression in the lung and intestine, low expression in the muscle, and abundance in the heart, stomach, kidney, spleen, liver, femur, skin and testis. Intermediate expression levels were observed in the brain and eye (Figure 3B,C). To ascertain the dynamics of KIAA0753 expression during osteoblast differentiation, we performed osteoblast differentiation induction on MC3T3‐E1 cells and bone marrow‐derived mesenchymal stem cells (BMSCs), respectively. Results demonstrated a continuous expression of KIAA0753 throughout the osteoblast differentiation process, with a notable increase in expression correlating with extended induction times (Figure 3D–G). These results suggested that the expression of KIAA0753 was elevated in a time‐dependent during osteoblast differentiation.

### 3.4 KIAA0753 rescues osteoblast differentiation inhibited by high glucose

**FIGURE 4:**
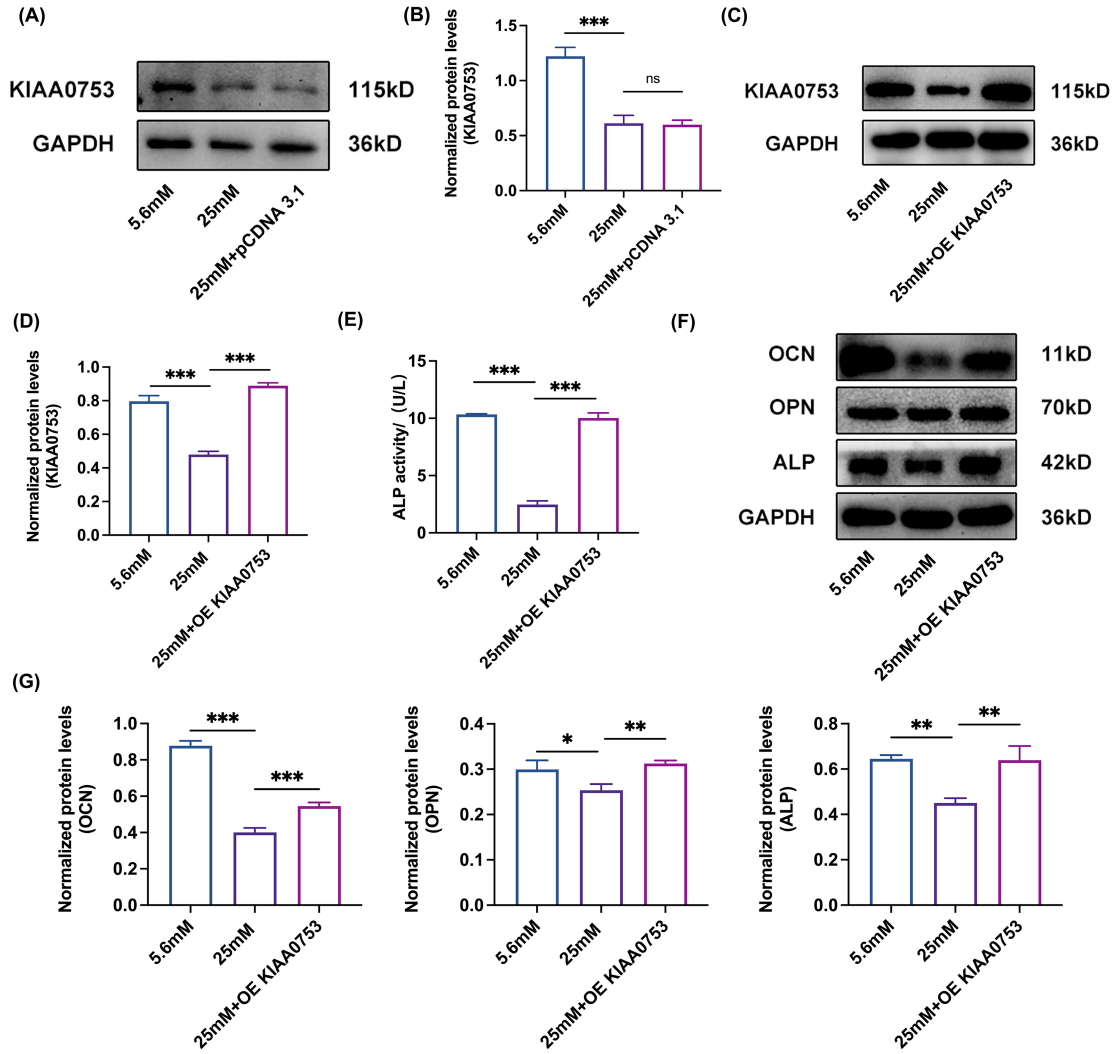
KIAA0753 rescues the osteoblast differentiation inhibited by high glucose. (A and B) Western blot of KIAA0753 expression in MC3T3‐E1 transfected with pcDNA 3.1‐Flag plasmids in high glucose for 3 days. (C and D) Western blot of KIAA0753 expression in MC3T3‐E1 transfected with pcDNA3.1‐KIAA0753‐Flag plasmids in high glucose for 3 days. (E) ALP activity assay by ELISA kit in MC3T3‐E1 transfected with pcDNA3.1‐KIAA0753‐Flag plasmids in high glucose for 5 days. (F and G) Western blot of ALP, OPN, OCN expression in MC3T3‐E1 transfected with pcDNA3.1‐KIAA0753‐Flag plasmids in high glucose for 3 days. All the data were represented as means ± SEM. *p < 0.05, **p < 0.01, ***p < 0.001, ns, no significance (*n* = 3).

As high glucose led to KIAA0753 expression and osteoblast differentiation decreased, we further explored the links between KIAA0753 and osteoblast differentiation. Cell models of overexpressing KIAA0753 (OE KIAA0753) were constructed by transfecting MC3T3‐E1 with pcDNA3.1‐Flag or pcDNA3.1‐KIAA0753‐Flag in high glucose. Notably, KIAA0753 expression exhibited no significant difference in HG + pCDNA3.1‐Flag groups, indicating the plasmids have no experimental impact (Figure 4A,B). In contrast, KIAA0753 levels significantly increased in HG + OE KIAA0753 groups compared with HG groups (Figure 4C,D). Subsequent evaluation of ALP activity using ELISA kits revealed a higher ALP activity in HG + OE KIAA0753 groups compared with HG groups (Figure 4E). Furthermore, results also demonstrated the capacity of KIAA0753 enhanced the protein expression levels of OCN, OPN and ALP in high glucose (Figure 4F,G). These results suggested that KIAA0753 promoted osteoblast differentiation by augmenting the expression of key osteoblast differentiation proteins (OCN, OPN and ALP) under high glucose.

### 3.5 KIAA0753 extends the primary cilia impaired by high glucose

**FIGURE 5:**
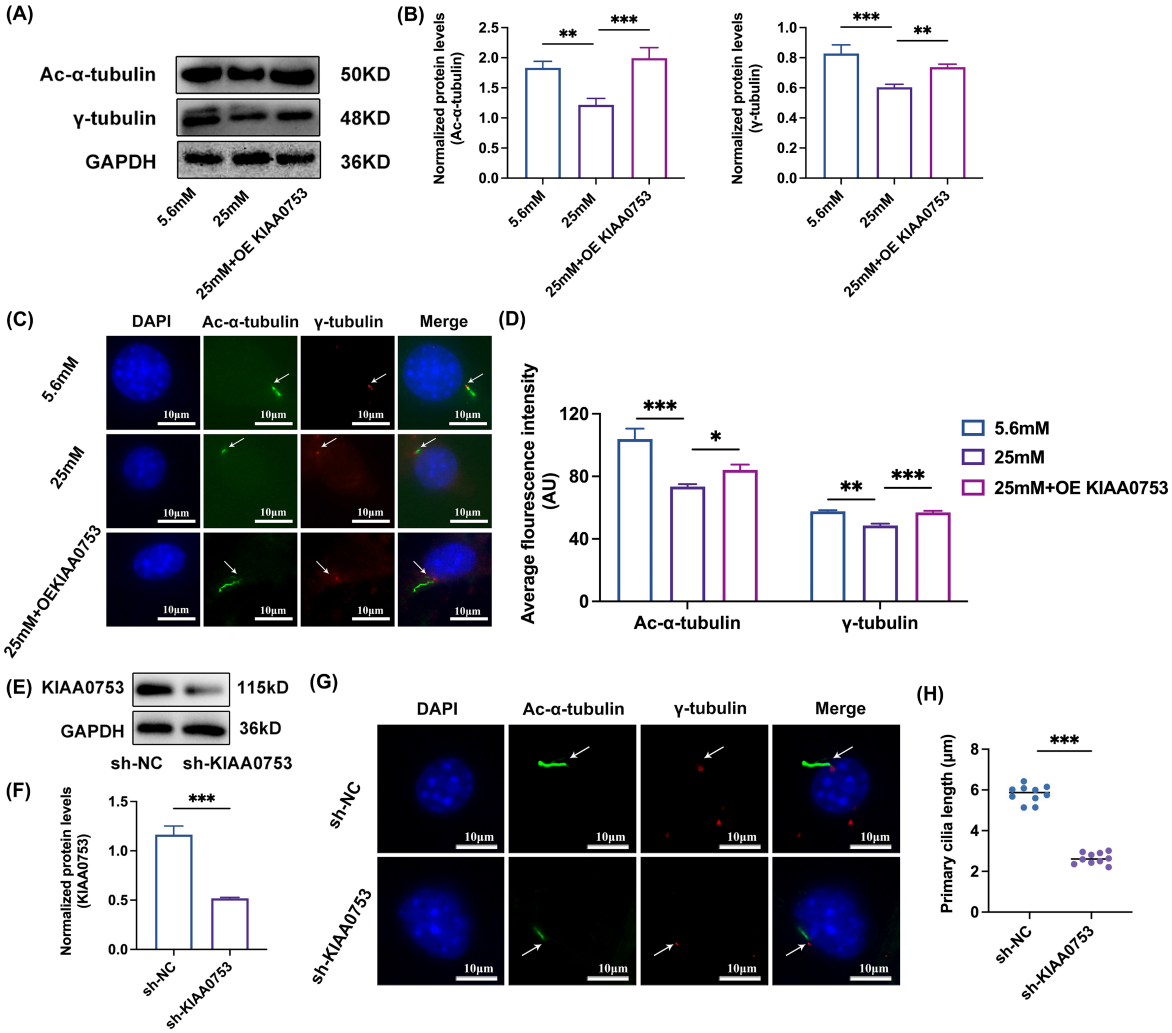
KIAA0753 extends the primary cilia impaired by high glucose. (A and B) Western blot of Ac‐α‐tubulin and γ‐tubulin expression in MC3T3‐E1 transfected with pcDNA3.1‐KIAA0753‐Flag plasmids in high glucose for 3 days. (C and D) Immunofluorescence staining of primary cilia in MC3T3‐E1 transfected with pcDNA3.1‐ KIAA0753‐Flag plasmids in high glucose for 3 days. Quantitative analysis of average fluorescence intensity of Ac‐α‐tubulin and γ‐tubulin. Blue, DAPI; Green, Ac‐α‐tubulin; Red, γ‐tubulin, scale bar: 10 μm. (E and F) Western blot of KIAA0753 expression in MC3T3‐E1 transfected with sh‐NC or sh‐KIAA0753 for 3 days. (G and H) Immunofluorescence staining of primary cilia in MC3T3‐E1 transfected with sh‐NC or sh‐KIAA0753 for 3 days. Quantitative analysis of primary cilia length. Blue, DAPI; Green, Ac‐α‐tubulin; Red, γ‐tubulin, scale bar: 10 μm. All the data were represented as means ± SEM. *p < 0.05, **p < 0.01, ***p < 0.001 (*n* ≥ 3).

To elucidate the potential correlation between increased osteoblast differentiation observed in MC3T3‐E1 cells overexpressing KIAA0753 and the state of primary cilia, we conducted a comprehensive examination of primary cilia alterations. The results demonstrated that KIAA0753 positively influenced the expression of Ac‐α‐tubulin and γ‐tubulin in high glucose (Figure 5A,B). Utilizing pcDNA3.1‐KIAA0753‐Flag plasmids for transfection in high glucose conditions, immunofluorescence staining revealed a significant extension of primary cilia length induced by KIAA0753 in high glucose (Figure 5C,D). To further assess the impact of KIAA0753 silencing on primary cilia, we employed lentivirus‐based RNAi gene silencing technology to knockdown KIAA0753 in MC3T3 cells. The following transfection with pLKO.1 puro‐KIAA0753 shRNA constructs, the recombinant lentiviruses were utilized to evaluate silencing efficiency. As depicted in Figure 5E,F, KIAA0753 protein expression was markedly reduced in sh‐KIAA0753 constructs compared with MC3T3‐E1 cells infected with the control pLKO.1 puro‐scrambled shRNA lentivirus. Immunofluorescence staining results further confirmed a significant reduction in primary cilia length after KIAA0753 knockdown (Figure 5G,H). Based on these findings, we postulated that KIAA0753 might promote osteoblast differentiation by safeguarding the functionality and morphology of primary cilia.

### 3.6 KIAA0753 protein interacts with Gli2, SHH and OCN to activate the Hedgehog signalling pathway inhibited by high glucose

**FIGURE 6:**
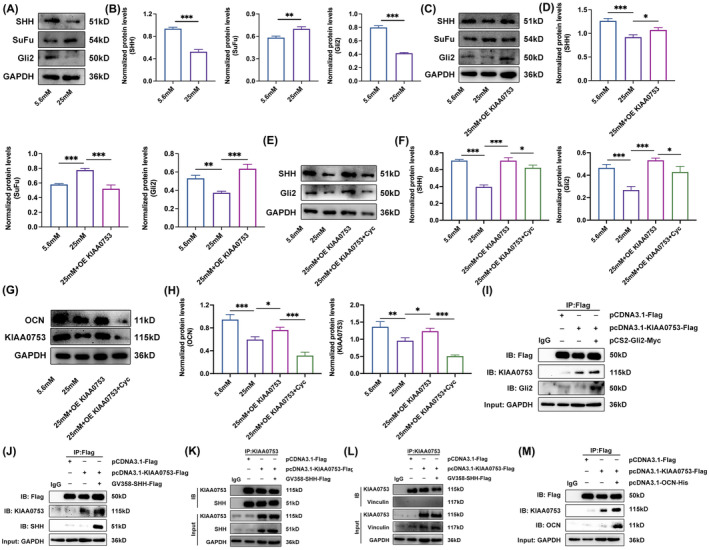
KIAA0753 protein interacts with Gli2, SHH and OCN protein to activate the Hedgehog signalling pathway inhibited by high glucose. (A and B) Western blot of SHH, SuFu and Gli2 expression in MC3T3‐E1 treated with 5.6 or 25 mM OS media for 3 days. (C and D) Western blot of SHH, SuFu and Gli2 expression in MC3T3‐E1 transfected with pcDNA3.1‐KIAA0753‐Flag plasmids in high glucose for 3 days. (E–H) Western blot of SHH, Gli2, KIAA0753 and OCN expression in MC3T3‐E1 transfected with pcDNA3.1‐KIAA0753‐Flag plasmids and incubated with 1 μmol/L Cyc in high glucose for 3 days. (I) Co‐IP of KIAA0753 with Gli2 in HEK293 T cells transfected with pcDNA3.1‐Flag, pcDNA3.1‐KIAA0753‐Flag or pCS2‐Gli2‐Myc for 48 h. (J and K) Co‐IP of KIAA0753 with SHH in HEK293 T cells transfected with pcDNA3.1‐Flag, pcDNA3.1‐KIAA0753‐Flag or GV358‐SHH‐Flag for 48 h. (L) Co‐IP of KIAA0753 with Vinculin in HEK293 T cells transfected with pcDNA3.1‐Flag, pcDNA3.1‐KIAA0753‐ Flag or GV358‐SHH‐Flag for 48 h. (M) Co‐IP of KIAA0753 with OCN in HEK293 T cells transfected with pcDNA3.1‐Flag, pcDNA3.1‐KIAA0753‐Flag or pcDNA3.1‐OCN‐His for 48 h. All the data were represented as means ± SEM. *p < 0.05, **p < 0.01, ***p < 0.001 (*n* = 3).

To investigate the potential involvement of the classic Hh signalling pathway in KIAA0753‐mediated promotion of osteoblast differentiation, we further studied the specific molecular mechanisms underlying KIAA0753's regulation of this pathway. The results revealed a decrease in SHH and Gli2 expression accompanied by an increase in SuFu expression in HG groups (Figure 6A,B). Conversely, overexpression of KIAA0753 in high glucose conditions enhanced SHH and Gli2 expression while inhibiting SuFu expression (Figure 6C,D). To determine the direct impact of KIAA0753 on the Hh signalling pathway, we performed an experiment in which MC3T3‐E1 cells were incubated with both overexpression KIAA0753 and cyclopamine (Cyc, a specific Hh signalling pathway inhibitor) in high glucose. The results demonstrated that Cyc significantly suppressed SHH and Gli2 expression in high glucose, while the effect was counteracted by overexpression KIAA0753 (Figure 6E,F). Additionally, the protein expressions of KIAA0753 and OCN were inhibited by Cyc in high glucose (Figure 6G,H). These results indicated that KIAA0753, at partially, directly activated the Hh signalling pathway to promote osteoblast differentiation. To further elucidate the regulatory mechanism of KIAA0753 on the Hh signalling pathway, coimmunoprecipitation (Co‐IP) experiments were conducted in HEK293T cells. The results showed that KIAA0753 interacted with both Gli2 and SHH (Figure 6I–K), with no interaction observed with Vinculin (a cytoplasmic actin‐binding protein) (Figure 6L). This indicated that KIAA0753 specifically interacted with Gli2 and SHH to directly regulate the Hh signalling pathway. Moreover, KIAA0753 was found to interact with OCN protein (Figure 6M). Collectively, these data suggested that the KIAA0753 protein, by forming complexes with SHH, Gli2 and OCN proteins, activated the Hh signalling pathway to improve osteoblast differentiation in the context of diabetes.

### 3.7 KIAA0753 inhibits ubiquitination promoted by high glucose and Gli2's ubiquitination

**FIGURE 7:**
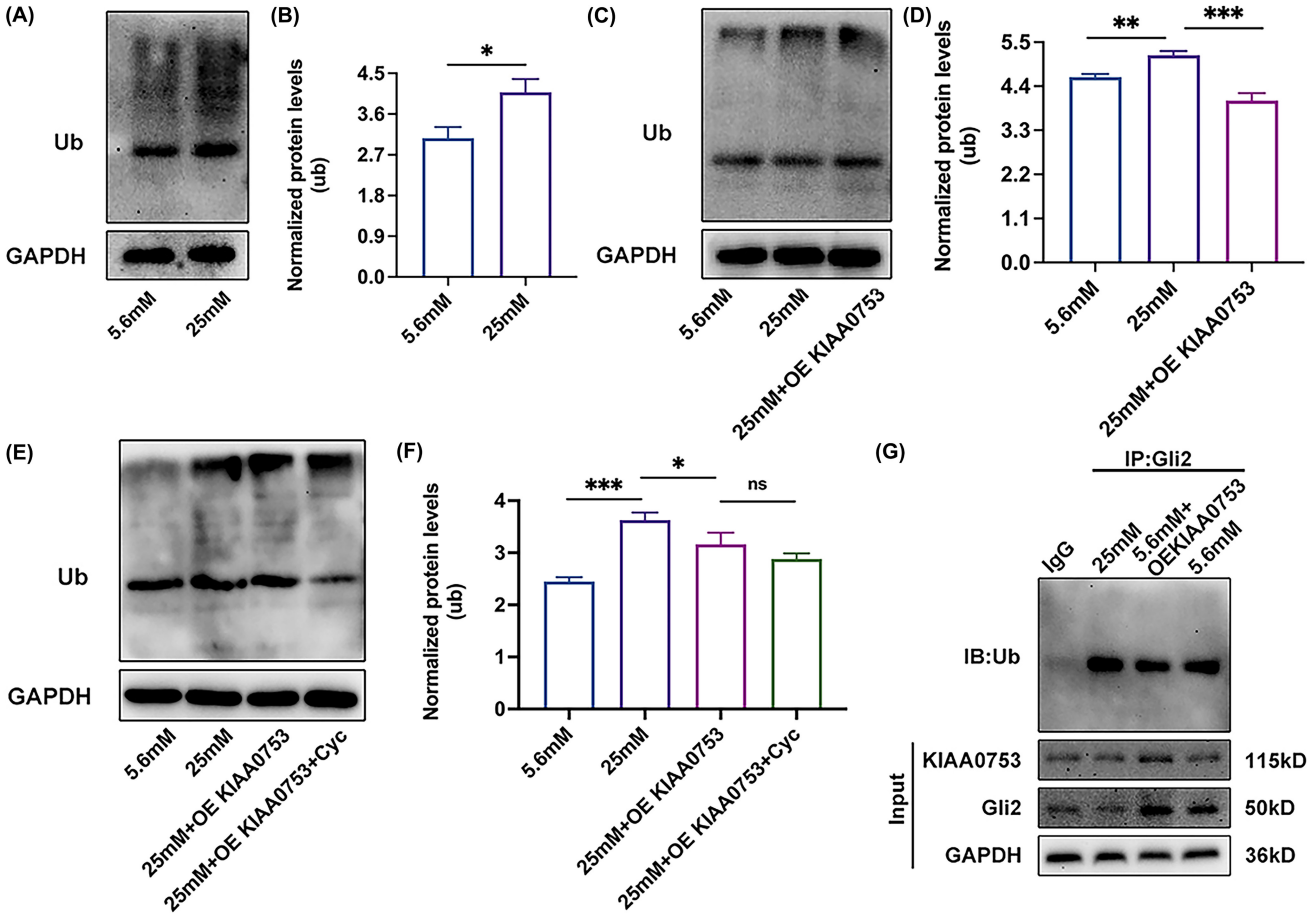
KIAA0753 inhibits ubiquitination promoted by high glucose and Gli2's ubiquitination. (A and B) Western blot of Ubiquitination protein expression in MC3T3‐E1 treated with 5.6 or 25 mM OS media for 3 days. (C and D) Western blot of Ubiquitination protein expression in MC3T3‐E1 transfected with pcDNA3.1‐ KIAA0753‐Flag plasmids in high glucose for 3 days. (E and F) Western blot of Ubiquitination protein expression in MC3T3‐E1 transfected with pcDNA3.1‐KIAA0753‐Flag plasmids and treated with 1 μmol/L Cyc in high glucose for 3 days. (G) Co‐IP of Gli2 with Ubiquitination protein in MC3T3‐E1 treated with 5.6, 25 or 5.6 mM + OE KIAA0753 for 3 days. All the data were represented as means ± SEM. *p < 0.05, **p < 0.01, ***p < 0.001, ns, no significance (*n* = 3).

To investigate the interplay between high glucose and ubiquitination, MC3T3‐E1 cells were induced by high glucose with or without overexpression of KIAA0753, followed by ubiquitination assay or Gli2 antibody pulldown assay. The analysis of total cell lysate proteins using a ubiquitination antibody revealed a significant increase in ubiquitination of MC3T3‐E1 cells under high glucose conditions compared with NC groups (Figure 7A,B). To further explore whether KIAA0753 has an effect on ubiquitination in high glucose, MC3T3‐E1 cells were transfected with pcDNA3.1‐KIAA0753‐Flag plasmids or treated with Cyc in high glucose. Results indicated that KIAA0753 inhibited ubiquitination in high glucose (Figure 7C,D). However, intriguingly, there were no differences in the protein expression levels of ubiquitination between the treatment with Cyc and OE KIAA0753 groups and OE KIAA0753 groups in high glucose (Figure 7E,F). This suggested that while KIAA0753 inhibited ubiquitination in high glucose to promote osteoblast differentiation, the ubiquitination process might also be regulated by other signalling pathways. Given that KIAA0753 interacted with the Gli2 protein, it remained unknown whether KIAA0753 played a role in the degradation process of the Gli2 protein. Co‐IP analysis was employed to assess their association, and the results demonstrated that high glucose promoted the ubiquitination of the Gli2 protein while overexpression of KIAA0753 reduced the ubiquitination of Gli2 protein (Figure 7G). These results suggested that KIAA0753 inhibited the ubiquitination of Gli2 protein promoted by high glucose. Consequently, Gli2, acting as a transcription factor, was translocated to activate the transcription of OCN, thereby upregulating the protein level of OCN and promoting osteoblast differentiation impaired by diabetes.

## 4 DISCUSSION

**FIGURE 8:**
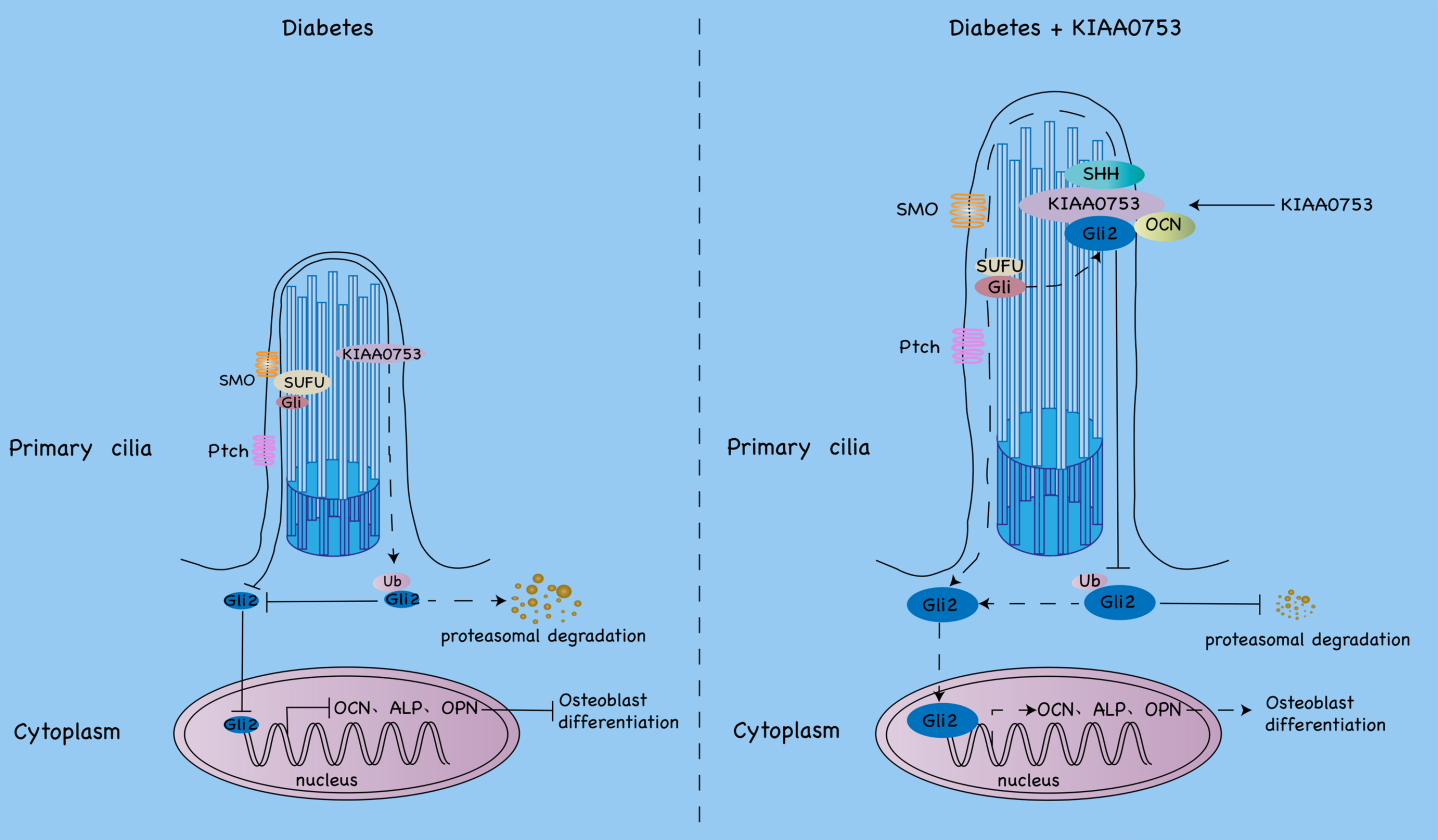
Proposed model of KIAA0753 promoted osteoblast differentiation by interacting with SHH, OCN and Gli2 to activate the Hh signalling pathway and decreasing the ubiquitination of Gli2 protein in diabetes.

Diabetes represents a serious threat to the health of humans, leading to many complications, such as retinopathy,^24^ diabetic nephropathy^25^ and so on. There are two types of diabetes, type 1 diabetes mellitus (T1DM) and type 2 diabetes mellitus (T2DM). Recently studies have shown that the bone mineral density (BMD) of T1DM patients is lower than healthy subjects, but T2DM patients have a higher BMD compared with healthy subjects.^26^, ^27^ They also suggest that T2DM accelerates bone loss at a faster speed than healthy subjects.^28^ Both of them have the same features that cause high glucose, promote AGEs formation, and generate ROS and inflammation.^29^ The generation of ROS and the activation of NF‐κB will affect osteoblast function and increase osteoclast formation, thereby adding bone resorption and bone loss.^30^ Diabetes‐induced hyperglycemia may promote proinflammatory cytokine expression, such as TNF‐α, leading to reduce osteoblast differentiation and increase osteoblast apoptosis.^31^ Similarly, diabetes reduces osteoblast differentiation by decreasing the expression of osteoblast marker genes.^32^ However, the effect of diabetes on osteoblast differentiation has not been well defined. This study for the first time confirmed that KIAA0753 could promote osteoblast differentiation by interacting with SHH and Gli2 to activate the Hh signalling pathway directly and decreasing the ubiquitination of Gli2 protein in diabetes (Figure 8).

KIAA0753, also known as MNR or OFIP, is a component of centrosome and pericentriolar satellites that can form complexes with other known satellite proteins, such as PCM1, OFD1 and FOR20.^33^ The process of centriole duplication is regulated by KIAA0753 through the recruitment of WDR62, CEP63 and CDK2 to the centrosome.^17^ Our results showed that KIAA0753 was high‐expressed in the lung and intestine and low‐expressed in the muscle. Moreover, it was found to be abundant in the heart, stomach, kidney, spleen, liver, femur, skin and testis. However, there was a middle expression level in the brain and eye. The expression of KIAA0753 had a significant decrease at the protein level in both diabetic cells and diabetic rats. Previous studies report that diabetes causes osteoporosis and fractures.^7^ Consistently, we found that high glucose inhibited osteoblast differentiation and the expression of KIAA0753 was increased in a time‐dependence pattern during osteoblast differentiation. Our results also suggested that KIAA0753 could rescue osteoblast differentiation in high glucose by increasing the expression of osteoblast‐specific proteins of OCN, OPN and ALP.

Recently studies have reported that mutations of KIAA0753 impair ciliogenesis to cause many skeletal ciliopathies, such as Joubert syndrome^34^ and oral‐facial‐digital syndrome (OFD).^35^ Ciliogenesis and maintenance of primary cilia rely on the interaction of the various components of molecular complexes, including OFIP‐OFD1‐FOR20 complexes.^18^ Thus, we speculate that KIAA0753 has some links with primary cilia. In this study, we confirmed that KIAA0753 was only distributed in the axis of primary cilia, not in the basal body. Recent studies have shown that loss of primary cilia increases diabetes susceptibility.^36^ Diabetes leads to a decrease in the number of ciliated beta cells as well as a dysregulation in the expression of ciliary genes,^37^ demonstrating the important role of primary cilia in the regulation of diabetes. Primary cilia serve as essential sensory organelles to sense and transduce extracellular signalling and thereby regulate cell behaviours.^38^ Primary cilia structural proteins and related accessory proteins are required for the integrity and function of primary cilia, and mutations in genes encoding these proteins lead to the degradation or dysfunction of primary cilia, resulting in diseases called ciliopathies.^15^ Although mutations of KIAA0753 caused skeletal ciliopathies have been reported, it is unknown whether KIAA0753 regulates primary cilia in diabetes. Our results suggested that high glucose inhibited the expression of primary cilia proteins Ac‐α‐tubulin and γ‐tubulin to impair primary cilia growth while KIAA0753 decreased the impaired by high glucose. We also found that the length of primary cilia was short significantly after KIAA0753 knockdown. These findings lent support to KIAA0753 might promote osteoblast differentiation by protecting the function and morphology of primary cilia.

Previous studies show that the Hh signalling pathway is required for bone development.^39^ Most Hh pathway components, including Patched (Ptch) 1/2, Smoothened (Smo), Gli1/2/3 and SuFu, are localized on the primary cilia.^40^, ^41^, ^42^ Ptch acts as a receptor of the Hh signalling pathway to inhibit the function of Smo that a seven‐pass transmembrane protein like G‐protein coupled receptors.^43^ The Hh signalling combines with Ptc to release Smo, then activates the downstream transcription factor Cubitus interrupt (Ci) to transcription.^44^ There are three Ci homologues in mammals, including Gli1, Gli2 and Gli3.^45^ Without Hh signalling, Ci exists in a cytoplasmic complex comprising Costal2 (Cos2)/Fused (Fu)/Suppressor of Fused (SuFu), and processes into a shorter transcriptional repressor. The activation of Smo inhibits Ci processing and allows the full‐ length Ci to enter the nucleus to activate the transcription of target genes.^46^ To address the specific molecular mechanisms that KIAA0753 regulates osteoblast differentiation, we further studied the link between KIAA0753 and the Hh signalling pathway. Our results suggested that high glucose inhibited the Hh signalling pathway by inhibiting the protein expression of SuFu, SHH and Gli2, but KIAA0753 could rescue the inhibitation to activate the Hh signalling pathway. We further found that Cyc suppressed the role of KIAA0753 in high glucose to inhibit the expression of SHH and Gli2 as well as KIAA053 and OCN. Our results also suggested that KIAA0753 interacted with SHH, Gli2 and OCN, not interact with Vinculin. These findings explained why KIAA0753 regulated the Hh signalling pathway directly to promote osteoblast differentiation in high glucose. All data indicated that KIAA0753 protein binding with SHH, Gli2 and OCN protein complexes activated the Hh signalling pathway to improve osteoblast differentiation inhibited by diabetes.

Previous reports reveal that mutations in the ciliary protein RPGRIP1L cause ciliary dysfunctions resulting in ciliopathies and RPGRIP1L deficiency leads to a decrease in proteasomal activity to promote ubiquitination.^47^ As we all know, ubiquitination is a universal way of endogenous protein degradation that is a process in which ubiquitin molecules modify the targets through an enzymatic reaction cascade to control protein function.^48^ Previous studies report that ubiquitination plays a critical role in regulating the processes of osteoarthritis (OA).^49^ It has been proved that RGS12 promotes OA by enhancing ubiquitination^50^ and inhibiting 26S proteasome and lysine‐48‐linked ubiquitination can decrease OA.^51^ One of the morphological features of OA is the chronic destruction of cartilage,^52^ so we suppose that ubiquitination may play an important role in diabetic bone loss. However, the ciliary protein of KIAA0753 whether related to ubiquitination is unknown. Our results showed that KIAA0753 inhibited ubiquitination promoted by high glucose. We further found that KIAA0753 interacted with Gli2 to inhibit the ubiquitination of Gli2 promoted by high glucose. These results indicated that KIAA0753 inhibited the ubiquitination of Gli2 promoted by high glucose to increase osteoblast differentiation. More interestingly, our results suggested that KIAA0753 promoted osteoblast differentiation by regulating the Hh signalling pathway in high glucose, still, the level of ubiquitination was no significant difference between OE KIAA0753 groups and Cyc with OE KIAA0753 groups in high glucose. These results also suggested a possibility that other signalling pathways might also regulate the process of ubiquitination. Further studies are needed to answer these questions.

In conclusion, we have shown for the first time that KIAA0753 rescues osteoblast differentiation and primary cilia growth in diabetes by interacting with SHH and Gli2 to activate the Hh signalling pathway and repressing the ubiquitination of Gli2 protein in diabetes. Our ongoing study aims to conduct a deeper exploration of diabetes bone loss pathogenesis and provide a better strategy for treating bone loss induced by diabetes.

## AUTHOR CONTRIBUTIONS

**Mengxue Li:** Data curation (equal); investigation (equal); methodology (equal); writing – original draft (equal). **Yongqin Wang:** Data curation (equal); investigation (equal); writing – original draft (equal). **Xiangmei Wu:** Data curation (equal); software (equal). **Quanmei Chen:** Data curation (equal); validation (equal). **Jianguo Huang:** Formal analysis (equal); methodology (equal). **Huifang Zhu:** Resources (equal); software (equal). **Shengyong Yang:** Software (equal); validation (equal). **Jichun Wang:** Investigation (equal); resources (equal); visualization (equal). **Le Tai Li:** Investigation (equal); methodology (equal). **Xianjun Liu:** Validation (equal); visualization (equal). **Kang Fu:** Data curation (equal); software (equal). **Fangzhou Song:** Investigation (equal); resources (equal); software (equal); writing – review and editing (equal). **Changdong Wang:** Conceptualization (equal); investigation (equal).

## CONFLICT OF INTEREST STATEMENT

The authors declare that they have no known competing financial interests or personal relationships that could have appeared to influence the work reported in this paper.

## Data Availability

Data will be made available on request.
